# Supplementary material for: Mechanistic insights into the R-loop formation and cleavage in CRISPR-Cas12i1
Source: Nat Commun. 2021 Jun 9;12:3476. doi: 10.1038/s41467-021-23876-5 (PMC8190297; doi:10.1038/s41467-021-23876-5)
Supplement: Supplementary file 1 — SUPPLEMENTARY INFORMATION [file 41467_2021_23876_MOESM1_ESM.pdf]

SUPPLEMENTARY INFORMATION

Mechanistic insights into the R-loop formation and cleavage in CRISPR-Cas12i1

B. Zhang, D. Luo, Y. Li et al.

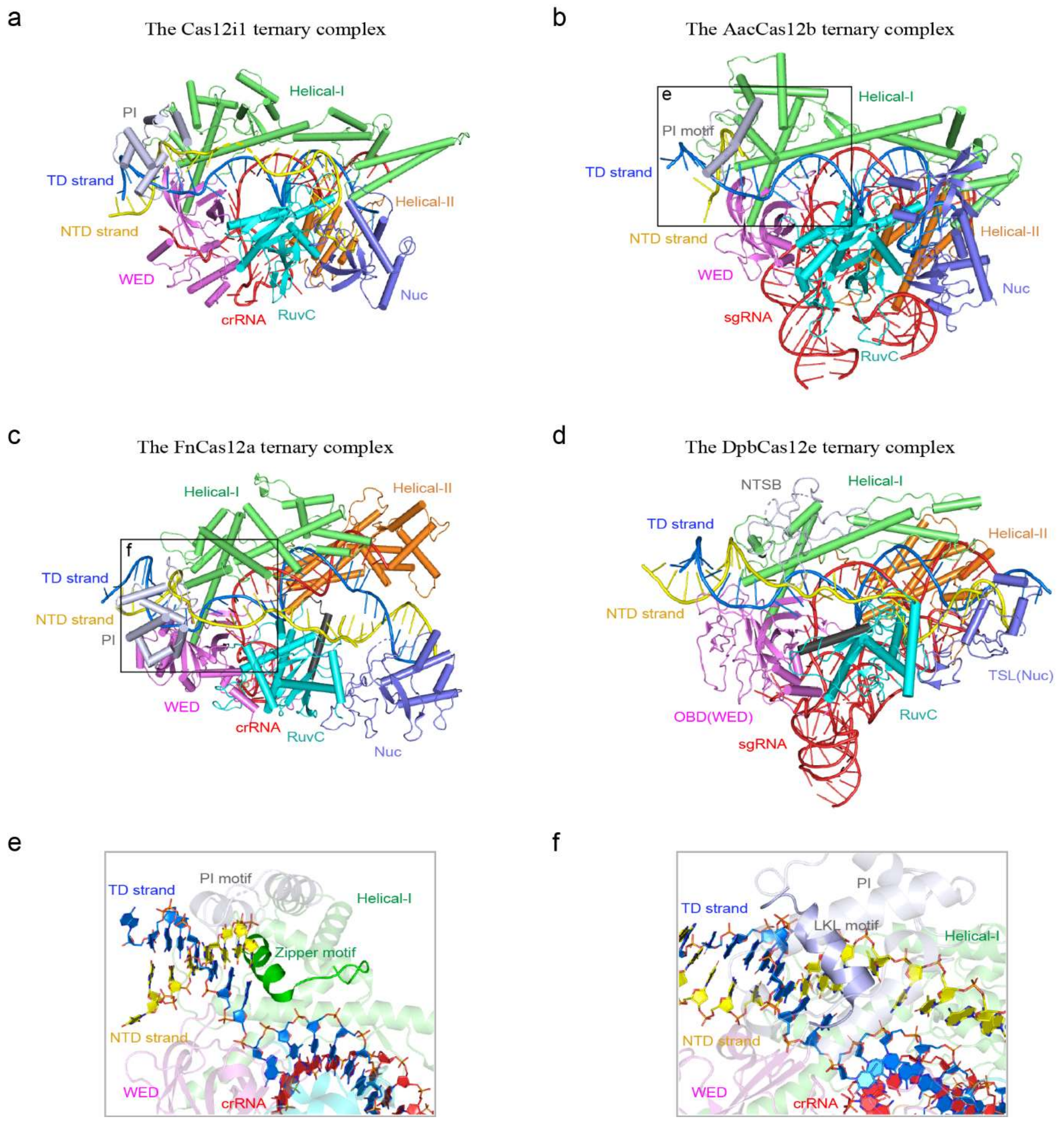

Supplementary Fig. 1 Comparison of the Cas12i1, AacCas12b, FnCas12a and DpbCas12e ternary complexes. a, Domain architecture of the Cas12i1 pre-cleavage R-loop complex (PDB code: 7D2L [https://doi.org/10.2210/pdb7D2L/pdb]). **b**, Domain architecture of the AacCas12b (*Alicyclobacillus acidoterrestris* Cas12b) ternary complex (PDB code: 5U30 [https://doi.org/10.2210/pdb5U30/pdb]). **c**, Domain architecture of the FnCas12a (*Francisella novicida* Cas12a) ternary complex (PDB code: 6I1K [https://doi.org/10.2210/pdb6I1K/pdb]). **d**, Domain architectures of the DpbCas12e (*Deltaproteobacteria* Cas12e) ternary complex (PDB code: 6NY2 [https://doi.org/10.2210/pdb6NY2/pdb]). **e**, Putative zipper motif in the AacCas12b ternary

complex may facilitate the target duplex unwinding. **f**, The LKL motif in the FnCas12a ternary complex facilitates the target duplex unwinding.

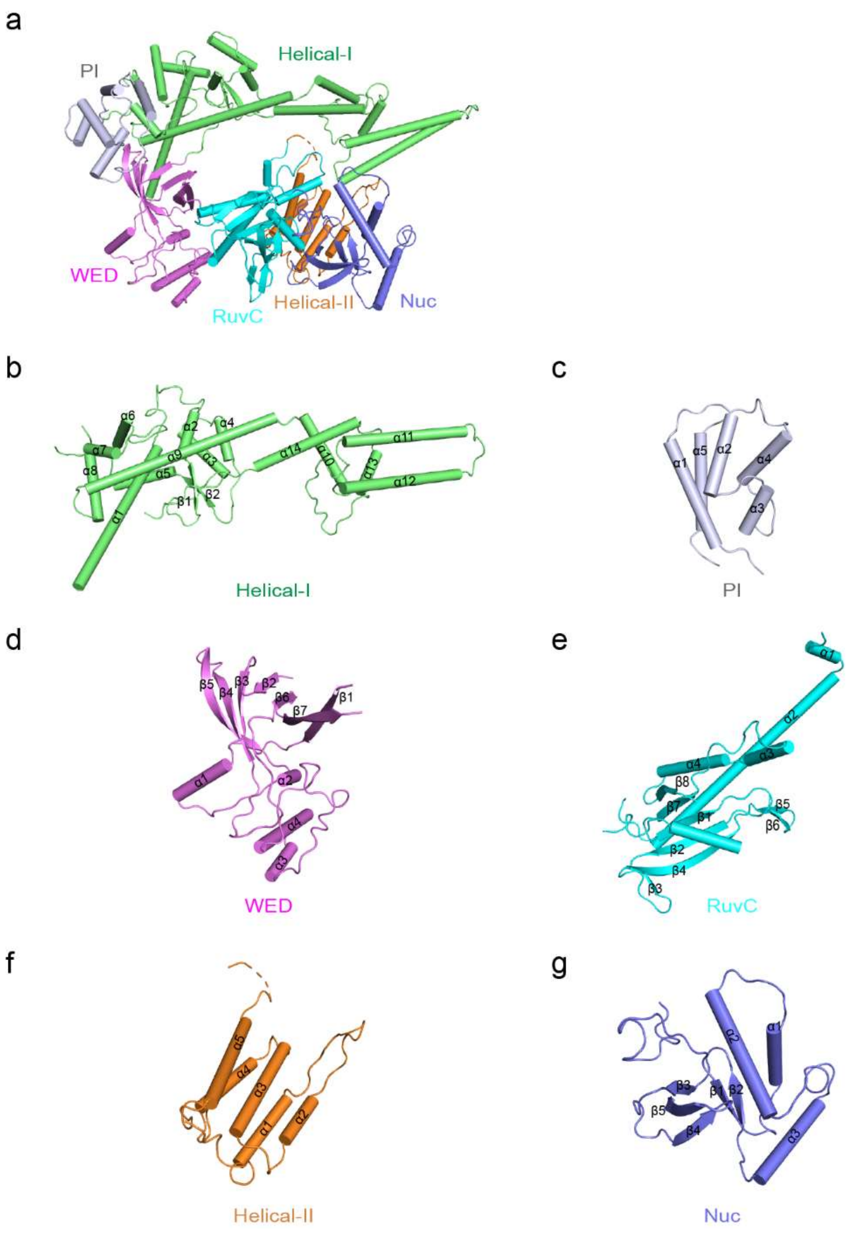

Supplementary Fig. 2 Structures of individual domains of Cas12i1 in the pre-cleavage R-loop complex. a, Cas12i1 structure in the Cas12i1 pre-cleavage R-loop complex. **b-g**, Structures of Helical-I, PI, WED, RuvC, Helical-II and Nuc domains in the Cas12i1 pre-cleavage R-loop complex. Domains are colored according to Fig. 1.

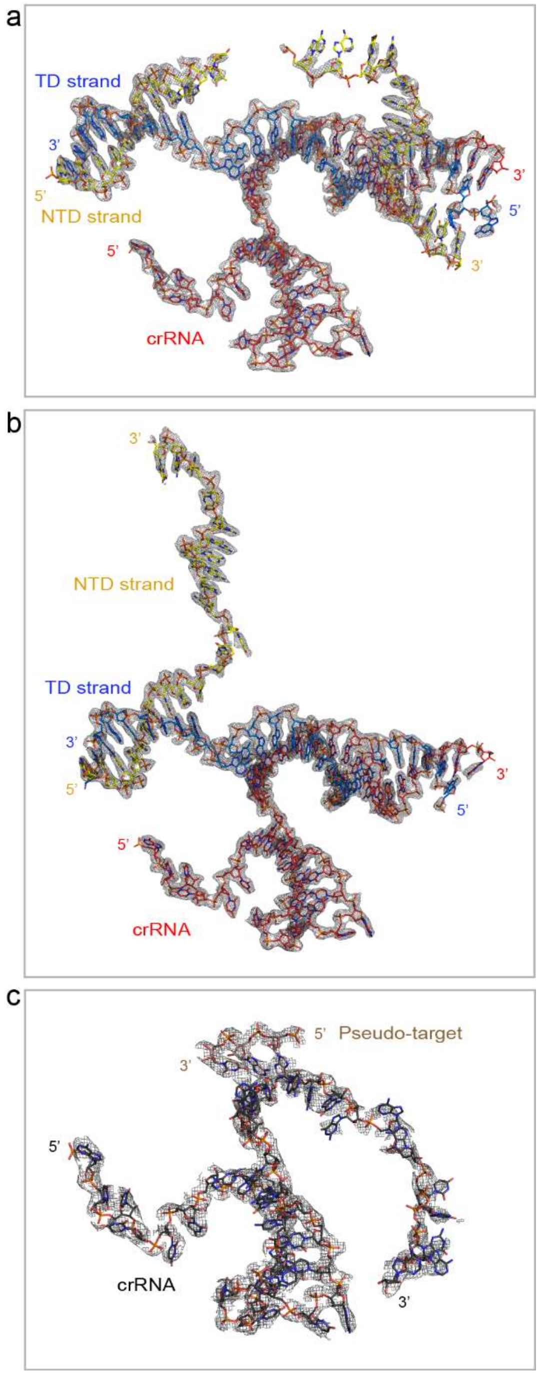

Supplementary Fig. 3 Electron density maps of nucleotides in the Cas12i1 binary and R-loop ternary complexes. a, The electron density map of crRNA, NTD and TD strands in the Cas12i1 pre-cleavage R-loop complex. **b**, The electron density map of crRNA, NTD and TD strands in the Cas12i1 post-cleavage R-loop complex. **c**, The electron density map of crRNA and the 3-nt pseudo target in the Cas12i1 binary complex. The 2Fo-Fc electron density maps were contoured at 1.0 σ level.

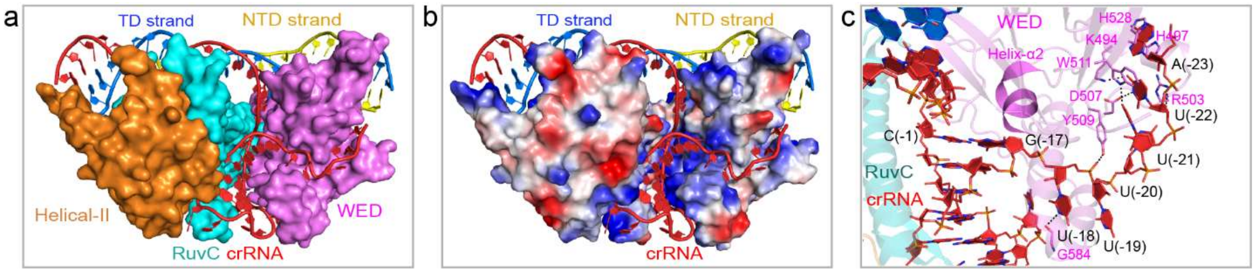

Supplementary Fig. 4 Recognition of the crRNA repeat region. a, The stem-loop structure of the crRNA repeat region occupies a groove formed by the WED, RuvC and Helical-II domains in the Cas12i1 pre-cleavage R-loop complex. **b**, Electrostatic potential surface of the crRNA-binding groove in the Cas12i1 pre-cleavage R-loop complex. Red, white and blue indicate negative, neutral and positive electrostatic potential surfaces, respectively. **c**, Detailed interactions between the nucleotides A(-23)–U(-18) of the crRNA repeat region and Cas12i1.

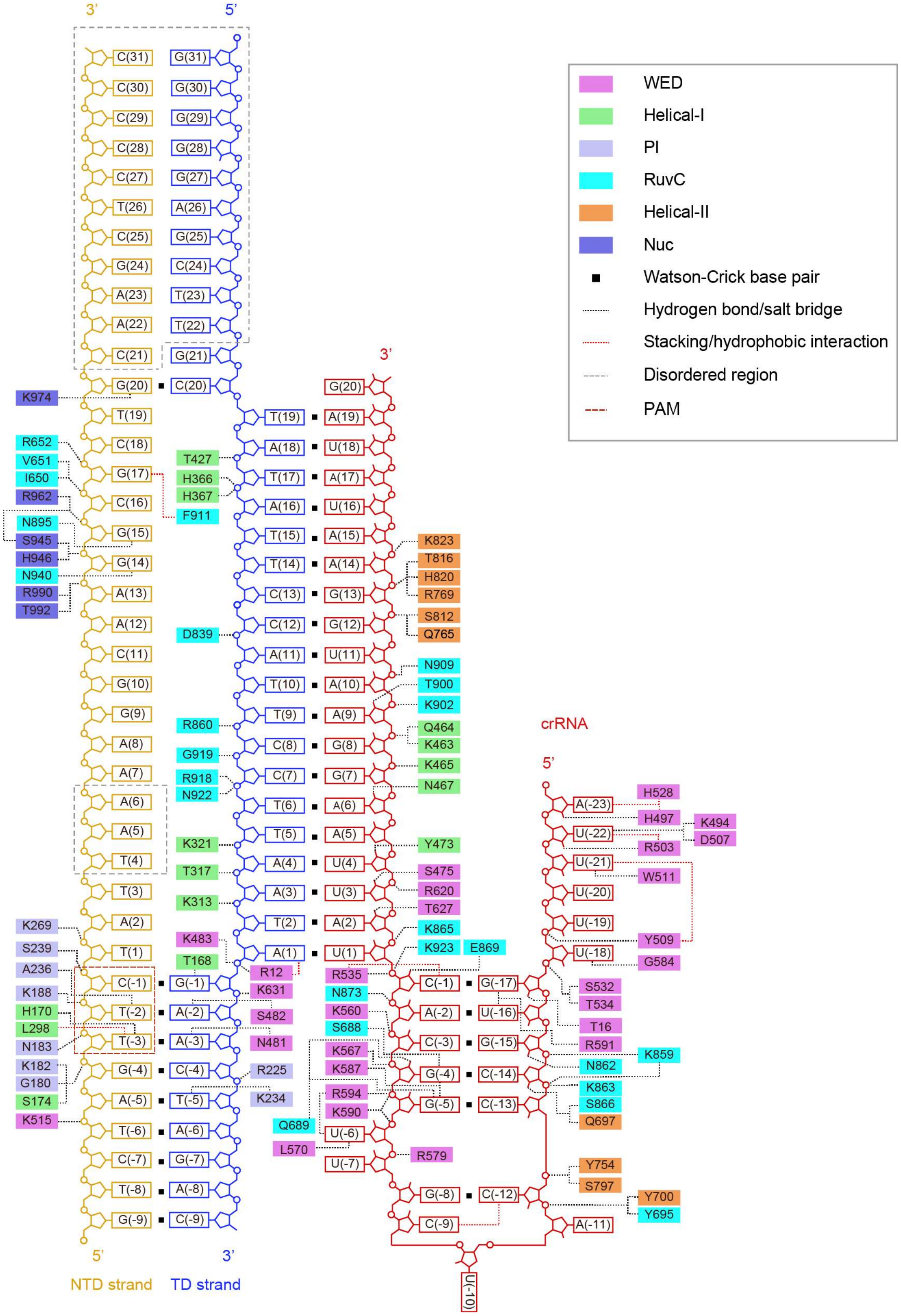

Supplementary Fig. 5 Schematic of the R-loop recognition in the Cas12i1 pre-cleavage R-loop complex. Domains and residues are colored according to Fig. 1. Hydrogen bonds and salt bridges are shown as black dashed lines. Stackings and hydrophobic interactions are shown as red dashed lines.

Disordered regions are encircled by gray dashed lines.

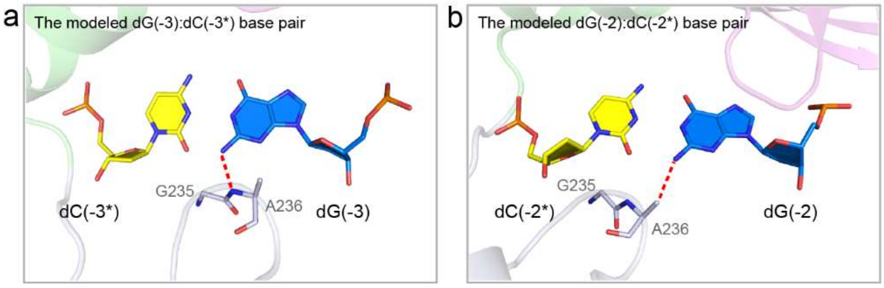

Supplementary Fig. 6 Recognition of the PAM duplex by Cas12i1. a, The modeled dG(-3):dC(-3*) base pair would generate a steric clash with the backbone nitrogen atom of residue A236. **b**, The modeled dG(-2):dC(-2*) base pair would generate a steric clash with the side chain of residue A236.

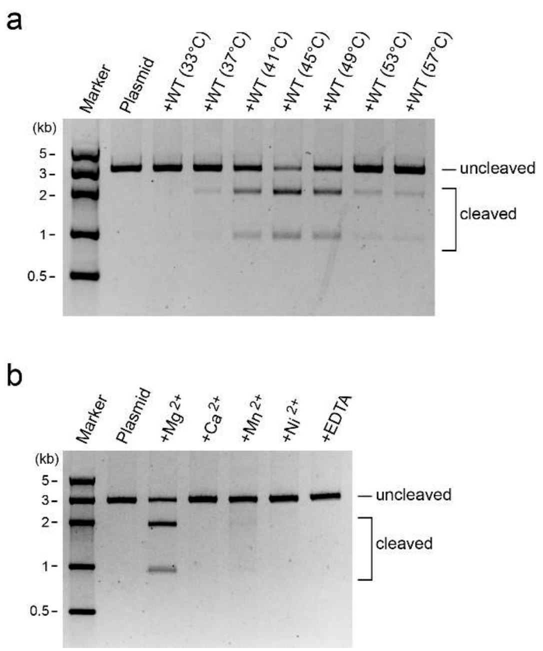

Supplementary Fig. 7 Temperature and metal-ion dependent cleavage of Cas12i1. a, Agarose gel demonstrating the cleavage of the linearized plasmid by wild-type Cas12i1 in complex with crRNA at different temperatures. **b**, Agarose gel demonstrating the cleavage of the linearized plasmid by wild-type Cas12i1 in complex with crRNA and in the presence of different divalent metal ions or EDTA. The cleavage assays of (**a**) and (**b**) were repeated three times independently to confirm the repeatability, respectively, and source data are provided as a Source Data file.

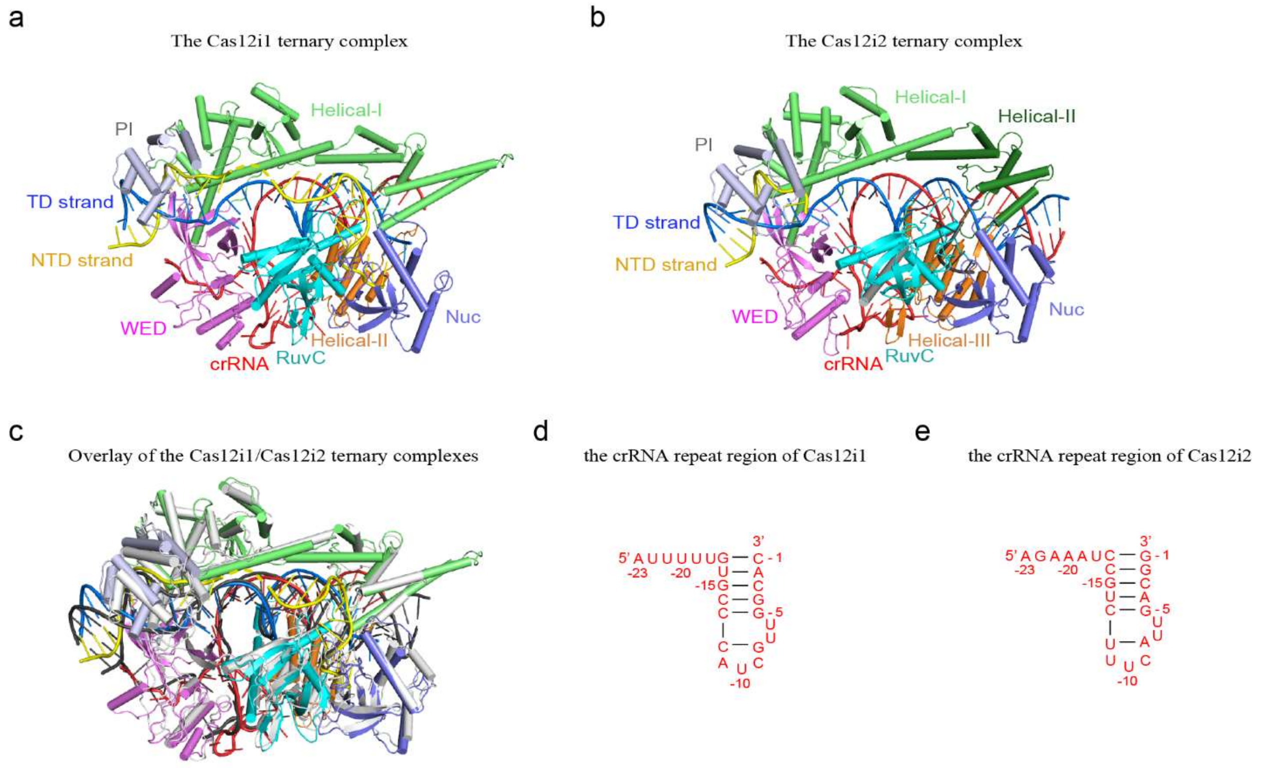

Supplementary Fig. 8 Comparison of the Cas12i1 and Cas12i2 ternary complexes. a, Domain architecture of the Cas12i1 pre-cleavage R-loop complex (PDB code: 7D2L [https://doi.org/10.2210/pdb7D2L/pdb]). **b**, Domain architecture of the Cas12i2 ternary complex (PDB code: 6LTR [https://doi.org/10.2210/pdb6LTR/pdb]). **c**, The overlay of the Cas12i1 pre-cleavage R-loop complex (PDB code: 7D2L [https://doi.org/10.2210/pdb7D2L/pdb]) and the Cas12i2 ternary complex (PDB code: 6LTR [https://doi.org/10.2210/pdb6LTR/pdb]). The Cas12i1 pre-cleavage R-loop complex is colored as in (**a**). Cas12i2 and nucleotides in the Cas12i2 ternary complex are colored in silver and black, respectively. **d**, Schematic representation of the crRNA repeat region in the CRISPR-Cas12i1 system. **e**, Schematic representation of the crRNA repeat region in the CRISPR-Cas12i2 system.

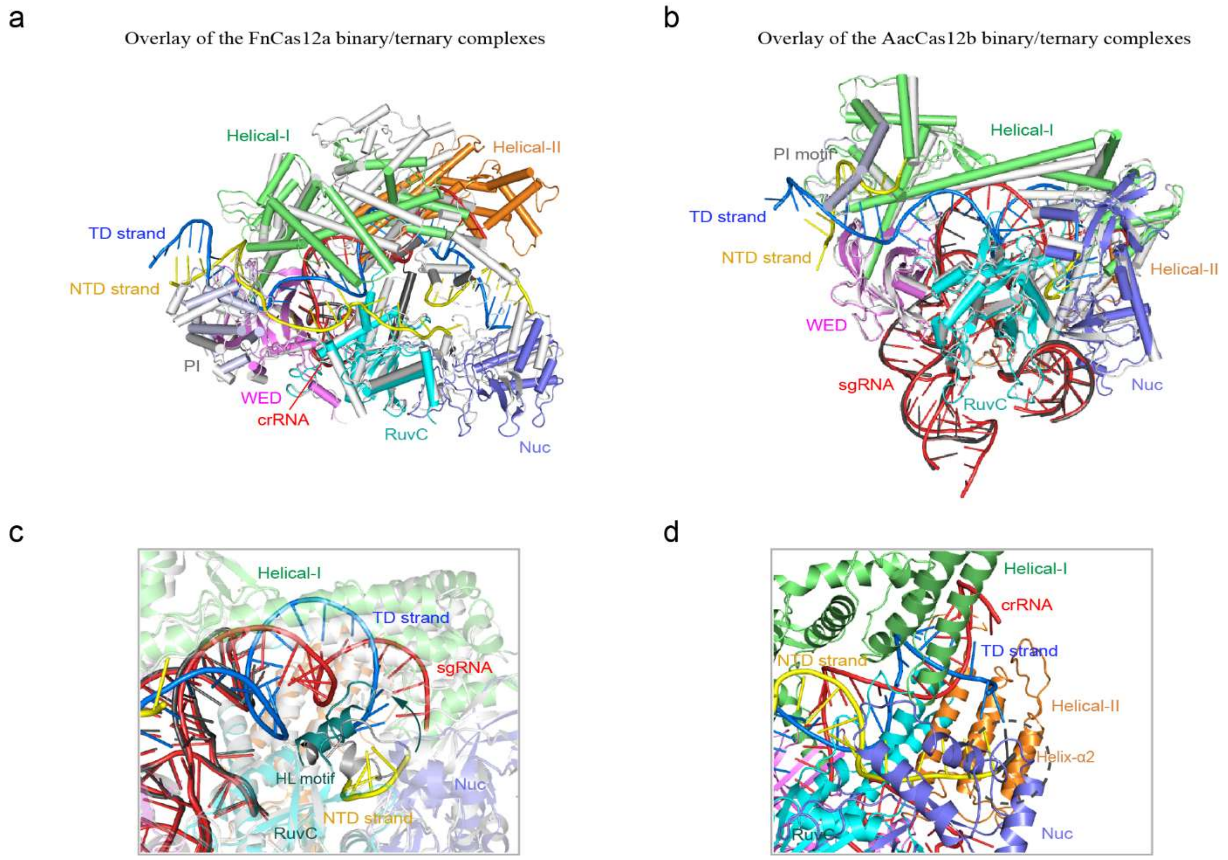

Supplementary Fig. 9 Conformational features of the FnCas12a, AacCas12b and Cas12i1 complexes. **a**, The overlay of the FnCas12a binary complex (PDB code: 5NG6 [https://doi.org/10.2210/pdb5NG6/pdb]) and the FnCas12a ternary complex (PDB code: 6I1K [https://doi.org/10.2210/pdb6I1K/pdb]). Domains, crRNA, NTD and TD strands of the FnCas12a ternary complex are colored and labeled. FnCas12a and crRNA of the FnCas12a binary complex are colored in silver and black, respectively. **b**, The overlay of the AacCas12b binary complex (PDB code: 5U34 [https://doi.org/10.2210/pdb5U34/pdb]) and the AacCas12b ternary complex (PDB code: 5U33 [https://doi.org/10.2210/pdb5U33/pdb]). Domains, crRNA, NTD and TD strands of the AacCas12b ternary complex are colored and labeled. AacCas12b and sgRNA of the AacCas12a binary complex are colored in silver and black, respectively. **c**, The overlay of the AacCas12b binary and ternary complexes displays the conformational rearrangement of the HL motif in its RuvC domain. **d**, A virtual-elongated 3′-end of the NTD strand in the Cas12i1 pre-cleavage R-loop complex clashes with helix α2 of the Helical-II domain. The virtual-elongated nucleotide is colored in black.

Supplementary Table 1 X-ray crystallography data collection and refinement statistics.

Dataset	SeMet-post-cleavage R-loop complex [a]	Pre-cleavage R-loop complex [b]	Post-cleavage R-loop complex [c]	Binary complex [d]
**Data collection**				
Space group	$C222_1$	$C222_1$	$C222_1$	$P6_322$
Cell dimensions				
a, b, c (Å)	130.58 142.93, 209.82	129.19, 142.64, 209.52	129.80, 141.97, 208.30	211.93, 211.93,164.76
α, β, γ (°)	90.0, 90.0, 90.0	90.0, 90.0, 90.0	90.0, 90.0, 90.0	90.0, 90.0, 120.0
Resolution range (Å) *	96.40-2.87 (2.94-2.87)	58.96-2.75 (2.84-2.75)	58.66-2.45 (2.51-2.45)	57.35-3.60 (3.85-3.60)
R_{merge}	0.155 (1.279)	0.070 (0.761)	0.083 (0.767)	0.327 (1.713)
$I/\sigma(I)$	15.3 (2.2)	24.3 (3.7)	17.6 (3.4)	12.3 (3.6)
Completeness (%)	99.5 (99.2)	100.0 (100.0)	100.0 (100.0)	99.9 (100.0)
Multiplicity	12.3 (13.8)	13.4 (14.1)	13.4 (13.9)	38.6 (38.6)
**Refinement**				
Resolution (Å)		2.75	2.45	3.60
No. unique reflections		50,543	70,800	25,821
R_{work} / R_{free} (%)		21.3/26.4	22.0/25.7	28.0/30.8
No. atoms				
Protein		8,684	8717	8557
Nucleic acid		2,060	1957	871
Ligand		13	13	13
Water		12	40	
B-factors (Å^2)				
Protein		76.83	61.95	144.67
Nucleic acid		84.60	62.40	143.25
Ligand		82.22	66.46	149.80
Water		56.99	43.25	
R.m.s deviations				
Bond lengths (Å)		0.009	0.009	0.006
Bond angles (°)		1.44	1.45	1.09
Ramachandran plot (%)				
Favored region		97.86	97.30	93.24
Allowed region		2.14	2.70	6.76
Outliers region		0	0	0

[a] The SeMet-labeled wild-type Cas12i1 post-cleavage R-loop complex.

[b] The catalytically inactive Cas12i1 (D647A) pre-cleavage R-loop complex (PDB code: 7D2L [https://doi.org/10.2210/pdb7D2L/pdb]).

[c] The wild-type Cas12i1 post-cleavage R-loop complex (PDB code: 7D3J [https://doi.org/10.2210/pdb7D3J/pdb]).

[d] The wild-type Cas12i1 binary complex (PDB code: 7D8C [https://doi.org/10.2210/pdb7D8C/pdb]).

*Highest resolution shell is shown in parentheses.

Supplementary Table 2 DNA coding sequence for Cas12i1 used in this study.

Description	DNA coding sequence*
Cas12i1	ATGAGCAACAAAGAAAAAAATGCAAGCGAAACCCGTAAAGCATATACCACCAAAATGATTCCG<br>CGTAGCCATGATCGTATGAAACTGCTGGGTAATTTTATGGATTATCTGATGGATGGTACCCCGAT<br>TTTTTTTGAACTGTGGAATCAGTTTGGTGGTGGTATTGATCGTGATATTATTAGCGGTACCGCAAA<br>TAAAGATAAAATTAGCGATGATCTGCTGCTGGCAGTTAATTGGTTTAAAGTTATGCCGATTAATA<br>GCAAACCGCAGGGTGTTAGCCCGAGCAATCTGGCAAATCTGTTTCAGCAGTATAGCGGTAGCGA<br>ACCGGATATTCAGGCACAGGAATATTTTGCAAGCAATTTTGATACCGAAAAACATCAGTGGAAA<br>GATATGCGTGTTGAATATGAACGTCTGCTGGCAGAACTGCAGCTGAGCCGTAGCGATATGCATCA<br>TGATCTGAAACTGATGTATAAAGAAAAATGTATTGGTCTGAGCCTGAGCACCGCACATTATATTA<br>CCAGCGTTATGTTTGGTACCGGTGCAAAAAATAATCGTCAGACCAAACATCAGTTTTATAGCAAA<br>GTTATTCAGCTGCTGGAAGAAAGCACCCAGATTAATAGCGTTGAACAGCTGGCAAGCATTATTCT<br>GAAAGCAGGTGATTGTGATAGCTATCGTAAACTGCGTATTCGTTGTAGCCGTAAAGGTGCAACCC<br>CGAGCATTCTGAAAATTGTTCAGGATTATGAACTGGGTACCAATCATGATGATGAAGTTAATGTT<br>CCGAGCCTGATTGCAAATCTGAAAGAAAAACTGGGTCGTTTTGAATATGAATGTGAATGGAAAT<br>GTATGGAAAAAATTAAAGCATTTCTGGCAAGCAAAGTTGGTCCGTATTATCTGGGTAGCTATAGC<br>GCAATGCTGGAAAATGCACTGAGCCCGATTAAAGGTATGACCACCAAAAATTGTAAATTTGTTCT<br>GAAACAGATTGATGCAAAAAATGATATTAAATATGAAAATGAACCGTTTGGTAAAATTGTTGAA<br>GGTTTTTTTGATAGCCCGTATTTTGAAAGCGATACCAATGTTAAATGGGTTCTGCATCCGCATCAT<br>ATTGGTGAAAGCAATATTAAAACCCTGTGGGAAGATCTGAATGCAATTCATAGCAAATATGAAG<br>AAGATATTGCAAGCCTGAGCGAAGATAAAAAAGAAAAACGTATTAAAGTTTATCAGGGTGATGT<br>TTGTCAGACCATTAATACCTATTGTGAAGAAGTTGGTAAAGAAGCAAAAACCCCGCTGGTTCAGC<br>TGCTGCGTTATCTGTATAGCCGTAAAGATGATATTGCAGTTGATAAAATTATTGATGGTATTACCT<br>TTCTGAGCAAAAAACATAAAGTTGAAAAACAGAAAATTAATCCGGTTATTCAGAAATATCCGAG<br>CTTTAATTTTGGTAATAATAGCAAACTGCTGGGTAAAATTATTAGCCCGAAAGATAAACTGAAAC<br>ATAATCTGAAATGTAATCGTAATCAGGTTGATAATTATATTTGGATTGAAATTAAAGTTCTGAAT<br>ACCAAAACCATGCGTTGGGAAAAACATCATTATGCACTGAGCAGCACCCGTTTTCTGGAAGAAG<br>TTTATTATCCGGCAACCAGCGAAAATCCGCCGGATGCACTGGCAGCACGTTTTCGTACCAAAACC<br>AATGGTTATGAAGGTAAACCGGCACTGAGCGCAGAACAGATTGAACAGATTCGTAGCGCACCGG<br>TTGGTCTGCGTAAAGTTAAAAAACGTCAGATGCGTCTGGAAGCAGCACGTCAGCAGAATCTGCT<br>GCCGCGTTATACCTGGGGTAAAGATTTTAATATTAATATTTGTAAACGTGGTAATAATTTTGAAG<br>TTACCCTGGCAACCAAAGTTAAAAAAAAAAAAGAAAAAAATTATAAAGTTGTTCTGGGTTATGA<br>TGCAAATATTGTTCGTAAAAATACCTATGCAGCAATTGAAGCACATGCAAATGGTGATGGTGTTA<br>TTGATTATAATGATCTGCCGGTTAAACCGATTGAAAGCGGTTTTGTTACCGTTGAAAGCCAGGTT<br>CGTGATAAAAGCTATGATCAGCTGAGCTATAATGGTGTTAAACTGCTGTATTGTAAACCGCATGT<br>TGAAAGCCGTCGTAGCTTTCTGGAAAAATATCGTAATGGTACCATGAAAGATAATCGTGGTAATA<br>ATATTCAGATTGATTTTATGAAAGATTTTGAAGCAATTGCAGATGATGAAACCAGCCTGTATTAT<br>TTTAATATGAAATATTGTAAACTGCTGCAGAGCAGCATTCGTAATCATAGCAGCCAGGCAAAAG<br>AATATCGTGAAGAAATTTTTGAACTGCTGCGTGATGGTAAACTGAGCGTTCTGAAACTGAGCAGC<br>CTGAGCAATCTGAGCTTTGTTATGTTTAAAGTTGCAAAAAGCCTGATTGGTACCTATTTTGGTCAT<br>CTGCTGAAAAAACCGAAAAATAGCAAAAGCGATGTTAAAGCACCGCCGATTACCGATGAAGATA<br>AACAGAAAGCAGATCCGGAAATGTTTGCACTGCGTCTGGCACTGGAAGAAAAACGTCTGAATAA<br>AGTTAAAAGCAAAAAAGAAGTTATTGCAAATAAAATTGTTGCAAAAGCACTGGAACTGCGTGAT<br>AAATATGGTCCGGTTCTGATTAAAGGTGAAAATATTAGCGATACCACCAAAAAAGGTAAAAAAA

	GCAGCACCAATAGCTTTCTGATGGATTGGCTGGCACGTGGTGTTGCAAATAAAGTTAAAGAAAT
	GGTTATGATGCATCAGGGTCTGGAATTTGTTGAAGTTAATCCGAATTTTACCAGCCATCAGGACC
	CTTTTGTTCATAAAAATCCGGAAAATACCTTTCGTGCACGTTATAGCCGTTGTACCCCGAGCGAA
	CTGACCGAAAAAAATCGTAAAGAAATTCTGAGCTTTCTGAGCGATAAACCGAGCAAACGTCCGA
	CCAATGCATATTATAATGAAGGTGCAATGGCATTTCTGGCAACCTATGGTCTGAAAAAAAATGAT
	GTTCTGGGTGTTAGCCTGGAAAAATTTAAACAGATTATGGCAAATATTCTGCATCAGCGTAGCGA
	AGATCAGCTGCTGTTTCCGAGCCGTGGTGGTATGTTTTATCTGGCAACCTATAAACTGGATGCAG
	ATGCAACCAGCGTTAATTGGAATGGTAAACAGTTTTGGGTTTGTAATGCAGATCTGGTTGCAGCA
	TATAATGTTGGTCTGGTTGATATTCAGAAAGATTTTAAAAAAAAAATAA

* Codons have been optimized for expression in *E. coli* Rosetta (DE3) cells.

Supplementary Table 3 DNA coding sequences for CRISPR array and crRNA used in the study.

Description	DNA coding sequences
CRISPR array	AATTTTTGTGCCCATCGTTGGCACTATTAAGGAATGGAATATAGCAAGCTAATTT<br>TTGTGCCCATCGTTGGCACTATTAAGGAATGGAATATAGCAAGCTAATTTTTGTG<br>CCCATCGTTGGCACTATTAAGGAATGGAATATAGCAAGCTAATTTTTGTGCCCAT<br>CGTTGGCACTATTAAGGAATGGAATATAGCAAGCTAATTTTTGTGCCCATCGTTG<br>GCACTATTAAGGAATGGAATATAGCAAGCTAATTTTTGTGCCCATCGTTGGCACT<br>ATTAAGGAATGGAATATAGCAAGCTAATTTTTGTGCCCATCGTTGGCACTATTAA<br>GGAATGGAATATAGCAAGCTAATTTTTGTGCCCATCGTTGGCAC
crRNA	AATTTTTGTGCCCATCGTTGGCACTATTAAGGAATGGAATATAGC

Supplementary Table 4 Primers used for Cas12i1 mutagenesis.

Description	Primers used for mutagenesis*
Cas12i1_R12A_F	AAAAATGCAAGCGAAACCGCAAAAGCATATACCACCAAA
Cas12i1_R12A_R	TTTGGTGGTATATGCTTTTGCGGTTTCGCTTGCATTTTT
Cas12i1_K13A_F	AATGCAAGCGAAACCCGTGCAGCATATACCACCAAAATG
Cas12i1_K13A_R	CATTTTGGTGGTATATGCTGCACGGGTTTCGCTTGCATT
Cas12i1_H170A_F	CTGAGCCTGAGCACCGCAGCATATATTACCAGCGTTATG
Cas12i1_H170A_R	CATAACGCTGGTAATATATGCTGCGGTGCTCAGGCTCAG
Cas12i1_S174A_F	ACCGCACATTATATTACCGCAGTTATGTTTGGTACCGGT
Cas12i1_S174A_R	ACCGGTACCAAACATAACTGCGGTAATATAATGTGCGGT
Cas12i1_G235A_F	ATTCGTTGTAGCCGTAAAGCAGCAACCCCGAGCATTCTG
Cas12i1_G235A_R	CAGAATGCTCGGGGTTGCTGCTTTACGGCTACAACGAAT
Cas12i1_A236L_F	CGTTGTAGCCGTAAAGGTCTGACCCCGAGCATTCTGAAA
Cas12i1_A236L_R	TTTCAGAATGCTCGGGGTCAGACCTTTACGGCTACAACG
Cas12i1_L298A_F	AAAGTTGGTCCGTATTATGCAGGTAGCTATAGCGCAATG
Cas12i1_L298A_R	CATTGCGCTATAGCTACCTGCATAATACGGACCAACTTT
Cas12i1_K313A_F	AATGCACTGAGCCCGATTGCAGGTATGACCACCAAAAAT
Cas12i1_K313A_R	ATTTTTGGTGGTCATACCTGCAATCGGGCTCAGTGCATT
Cas12i1_K318A_F	ATTAAAGGTATGACCACCGCAAATTGTAAATTTGTTCTG
Cas12i1_K318A_R	CAGAACAAATTTACAATTTGCGGTGGTCATACCTTTAAT
Cas12i1_K321A_F	ATGACCACCAAAAATTGTGCATTTGTTCTGAAACAGATT
Cas12i1_K321A_R	AATCTGTTTCAGAACAAATGCACAATTTTTGGTGGTCAT
Cas12i1_N481A_F	AGCTTTAATTTTGGTAATGCAAGCAAACTGCTGGGTAAA
Cas12i1_N481A_R	TTTACCCAGCAGTTTGCTTGCATTACCAAAATTAAAGCT
Cas12i1_S482A_F	TTTAATTTTGGTAATAATGCAAAACTGCTGGGTAAAATT
Cas12i1_S482A_R	AATTTTACCCAGCAGTTTTGCATTATTACCAAAATTAAA
Cas12i1_K483A_F	AATTTTGGTAATAATAGCGCACTGCTGGGTAAAATTATT
Cas12i1_K483A_R	AATAATTTTACCCAGCAGTGCGCTATTATTACCAAAATT
Cas12i1_R535A_F	TATGCACTGAGCAGCACCGCATTTCTGGAAGAAGTTTAT
Cas12i1_R535A_R	ATAAACTTCTTCCAGAAATGCGGTGCTGCTCAGTGCATA
Cas12i1_R620A_F	AATATTAATATTTGTAAAGCAGGTAATAATTTTGAAGTT
Cas12i1_R620A_R	AACTTCAAAATTATTACCTGCTTTACAAATATTAATATT
Cas12i1_K631A_F	GAAGTTACCCTGGCAACCGCAGTTAAAAAAAAAAAAGAA
Cas12i1_K631A_R	TTCTTTTTTTTTTTTAACTGCGGTTGCCAGGGTAACTTC
Cas12i1_D647A_F	AAAGTTGTTCTGGGTTATGCAGCAAATATTGTTCGTAAA
Cas12i1_D647A_R	TTTACGAACAATATTTGCTGCATAACCCAGAACAACTTT
Cas12i1_N649A_F	GTTCTGGGTTATGATGCAGCAATTGTTCGTAAAAATACC
Cas12i1_N649A_R	GGTATTTTTACGAACAATTGCTGCATCATAACCCAGAAC
Cas12i1_R652A_F	TATGATGCAAATATTGTTGCAAAAAATACCTATGCAGCA
Cas12i1_R652A_R	TGCTGCATAGGTATTTTTTGCAACAATATTTGCATCATA
Cas12i1_R860A_F	CTGGCACTGGAAGAAAAAGCACTGAATAAAGTTAAAAGC
Cas12i1_R860A_R	GCTTTTAACTTTATTCAGTGCTTTTTCTTCCAGTGCCAG
Cas12i1_K865A_F	AAACGTCTGAATAAAGTTGCAAGCAAAAAAGAAGTTATT
Cas12i1_K865A_R	AATAACTTCTTTTTTGCTTGCAACTTTATTCAGACGTTT

Cas12i1_E894A_F	CCGGTTCTGATTAAAGGTGCAAATATTAGCGATACCACC
Cas12i1_E894A_R	GGTGGTATCGCTAATATTTGCACCTTTAATCAGAACCGG
Cas12i1_W915A_F	AATAGCTTTCTGATGGATGCACTGGCACGTGGTGTTGCA
Cas12i1_W915A_R	TGCAACACCACGTGCCAGTGCATCCATCAGAAAGCTATT
Cas12i1_R918A_F	CTGATGGATTGGCTGGCAGCAGGTGTTGCAAATAAAGTT
Cas12i1_R918A_R	AACTTTATTTGCAACACCTGCTGCCAGCCAATCCATCAG
Cas12i1_K923A_F	GCACGTGGTGTTGCAAATGCAGTTAAAGAAATGGTTATG
Cas12i1_K923A_R	CATAACCATTTCTTTAACTGCATTTGCAACACCACGTGC
Cas12i1_S945A_F	GTTAATCCGAATTTTACCGCACATCAGGACCCTTTTGTT
Cas12i1_S945A_R	AACAAAAGGGTCCTGATGTGCGGTAAAATTCGGATTAAC
Cas12i1_H946A_F	AATCCGAATTTTACCAGCGCACAGGACCCTTTTGTTCAT
Cas12i1_H946A_R	ATGAACAAAAGGGTCCTGTGCGCTGGTAAAATTCGGATT
Cas12i1_R962A_F	GAAAATACCTTTCGTGCAGCATATAGCCGTTGTACCCCG
Cas12i1_R962A_R	CGGGGTACAACGGCTATATGCTGCACGAAAGGTATTTTC
Cas12i1_D1074A_F	TTTTGGGTTTGTAATGCAGCACTGGTTGCAGCATATAAT
Cas12i1_D1074A_R	ATTATATGCTGCAACCAGTGCTGCATTACAAACCCAAAA

* Forward and reverse primers are indicated by “F” and “R”, respectively.

Supplementary Table 5 Oligonucleotide used for cleavage assays.

Description	Oligonucleotide used for cleavage assays*
TD strand for cleavage assay	GGGGGAGCTTGCTATATTCCATTCCTTAATAGAACTAGAC
NTD Strand for cleavage assay	GTCTAGTTCTATTAAGGAATGGAATATAGCAAGCTCCCCC
TD strand for crystallization	GGGGGAGCTTGCTATATTCCATTCCTTAATAGAACTAGAC
NTD Strand for crystallization	GTCTAGTTCTATTAAAAGGCAAGGCGCTGCAAGCTCCCCC
3′-6FAM-TD strand	GGGGGAGCTTGCTATATTCCATTCCTTAATAGAACTAGAC-6FAM-3′
5′-6FAM-NTD strand	5′-6FAM-GTCTAGTTCTATTAAGGAATGGAATATAGCAAGCTCCCCC
5′-6FAM-NTD strand-mismatch-1nt	5′-6FAM-GTCTAGTTCGATTAAGGAATGGAATATAGCAAGCTCCCCC
5′-6FAM-NTD strand-mismatch-2nt	5′-6FAM-GTCTAGTTCGCTTAAGGAATGGAATATAGCAAGCTCCCCC
5′-6FAM-NTD strand-mismatch-3nt	5′-6FAM-GTCTAGTTCGCGTAAGGAATGGAATATAGCAAGCTCCCCC
5′-6FAM-NTD strand-mismatch-4nt	5′-6FAM-GTCTAGTTCGCGGAAGGAATGGAATATAGCAAGCTCCCCC
5′-6FAM-ladder-20nt	5′-6FAM-GTCTAGTTCTATTAAGGAAT
5′-6FAM-ladder-21nt	5′-6FAM-GTCTAGTTCTATTAAGGAATG
5′-6FAM-ladder-22nt	5′-6FAM-GTCTAGTTCTATTAAGGAATGG
5′-6FAM-ladder-23nt	5′-6FAM-GTCTAGTTCTATTAAGGAATGGA
5′-6FAM-ladder-24nt	5′-6FAM-GTCTAGTTCTATTAAGGAATGGAA
5′-6FAM-ladder-25nt	5′-6FAM-GTCTAGTTCTATTAAGGAATGGAAT
5′-6FAM-ladder-26nt	5′-6FAM-GTCTAGTTCTATTAAGGAATGGAATA
3′-6FAM-ladder-30nt	CGATATAAGGTAAGGAATTATCTTGATCTG-6FAM-3′
3′-6FAM-ladder-31nt	ACGATATAAGGTAAGGAATTATCTTGATCTG-6FAM-3′
3′-6FAM-ladder-32nt	AACGATATAAGGTAAGGAATTATCTTGATCTG-6FAM-3′
3′-6FAM-ladder-33nt	GAACGATATAAGGTAAGGAATTATCTTGATCTG-6FAM-3′
3′-6FAM-ladder-34nt	CGAACGATATAAGGTAAGGAATTATCTTGATCTG-6FAM-3′
3′-6FAM-ladder-35nt	TCGAACGATATAAGGTAAGGAATTATCTTGATCTG-6FAM-3′
5′-6FAM-non-specific-ssDNA-50nt	5′-6FAM-ATATATATATATATATATATATATATATATATATATATATATATATATAT
dsDNA-activator-NTD strand-20nt	GTCTAGTTCTATTAAGGAAT
dsDNA-activator-TD strand-20nt	ATTCCTTAATAGAACTAGAC
dsDNA-activator-NTD strand-21nt	GTCTAGTTCTATTAAGGAATG
dsDNA-activator-TD strand-21nt	CATTCCTTAATAGAACTAGAC
dsDNA-activator-NTD strand-22nt	GTCTAGTTCTATTAAGGAATGG
dsDNA-activator-TD strand-22nt	CCATTCCTTAATAGAACTAGAC
dsDNA-activator-NTD strand-23nt	GTCTAGTTCTATTAAGGAATGGA
dsDNA-activator-TD strand-23nt	TCCATTCCTTAATAGAACTAGAC
dsDNA-activator-NTD strand-24nt	GTCTAGTTCTATTAAGGAATGGAA
dsDNA-activator-TD strand-24nt	TTCCATTCCTTAATAGAACTAGAC
dsDNA-activator-NTD strand-25nt	GTCTAGTTCTATTAAGGAATGGAAT
dsDNA-activator-TD strand-25nt	ATTCCATTCCTTAATAGAACTAGAC
ssDNA-activator-11nt	ATTCCTTAATA
ssDNA-activator-12nt	CATTCCTTAATA
ssDNA-activator-13nt	CCATTCCTTAATA
ssDNA-activator-14nt	TCCATTCCTTAATA
ssDNA-activator-15nt	TTCCATTCCTTAATA
ssDNA-activator-16nt	ATTCCATTCCTTAATA

*The mutational nucleotides are colored in red.
