# Supplementary material for: Mechanistic insights into the R-loop formation and cleavage in CRISPR-Cas12i1
Source: Nat Commun. 2021 Jun 9;12:3476. doi: 10.1038/s41467-021-23876-5 (PMC8190297; doi:10.1038/s41467-021-23876-5)
Supplement: Supplementary file 3 — Reporting Summary [file 41467_2021_23876_MOESM3_ESM.pdf]

nature research

Corresponding author(s): Songying Ouyang
Last updated by author(s): May 14, 2021

Reporting Summary

Nature Research wishes to improve the reproducibility of the work that we publish. This form provides structure for consistency and transparency in reporting. For further information on Nature Research policies, see our Editorial Policies and the Editorial Policy Checklist.

Statistics

For all statistical analyses, confirm that the following items are present in the figure legend, table legend, main text, or Methods section.

n/a	Confirmed	
☐	☒	The exact sample size (*n*) for each experimental group/condition, given as a discrete number and unit of measurement
☐	☒	A statement on whether measurements were taken from distinct samples or whether the same sample was measured repeatedly
☒	☐	The statistical test(s) used AND whether they are one- or two-sided<br>*Only common tests should be described solely by name; describe more complex techniques in the Methods section.*
☒	☐	A description of all covariates tested
☒	☐	A description of any assumptions or corrections, such as tests of normality and adjustment for multiple comparisons
☒	☐	A full description of the statistical parameters including central tendency (e.g. means) or other basic estimates (e.g. regression coefficient) AND variation (e.g. standard deviation) or associated estimates of uncertainty (e.g. confidence intervals)
☒	☐	For null hypothesis testing, the test statistic (e.g. *F*, *t*, *r*) with confidence intervals, effect sizes, degrees of freedom and *P* value noted<br>*Give P values as exact values whenever suitable.*
☒	☐	For Bayesian analysis, information on the choice of priors and Markov chain Monte Carlo settings
☒	☐	For hierarchical and complex designs, identification of the appropriate level for tests and full reporting of outcomes
☒	☐	Estimates of effect sizes (e.g. Cohen's *d*, Pearson's *r*), indicating how they were calculated

Our web collection on statistics for biologists contains articles on many of the points above.

Software and code

Policy information about availability of computer code

Data collection	Blu-Ice
Data analysis	XIA2 (Version 0.5), XDS (Mar 15, 2019), PHENIX (Version 1.13-2998), and Coot (0.8.9) were used to analyze crystallographic data in this study. Autosol and Phaser-MR utilized in this study are parts of the PHENIX platform. AIMLESS and Refmac5 utilized in this study are parts of the CCP4 (Version 7.0.056) platform. Structures were visualized by PyMOL (Version 2.1.1, Educational subscriptions).

For manuscripts utilizing custom algorithms or software that are central to the research but not yet described in published literature, software must be made available to editors and reviewers. We strongly encourage code deposition in a community repository (e.g. GitHub). See the Nature Research guidelines for submitting code & software for further information.

Data

Policy information about availability of data

All manuscripts must include a data availability statement. This statement should provide the following information, where applicable:

- Accession codes, unique identifiers, or web links for publicly available datasets
- A list of figures that have associated raw data
- A description of any restrictions on data availability

The corresponding models in the Protein Data Bank under the accession codes 7D2L [https://doi.org/10.2210/pdb7D2L/pdb], 7D3J [https://doi.org/10.2210/pdb7D3J/pdb] and 7D8C [https://doi.org/10.2210/pdb7D8C/pdb]. The manuscript includes a Data availability statement that containing this information.

Field-specific reporting

Please select the one below that is the best fit for your research. If you are not sure, read the appropriate sections before making your selection.

☒ Life sciences ☐ Behavioural & social sciences ☐ Ecological, evolutionary & environmental sciences

For a reference copy of the document with all sections, see nature.com/documents/nr-reporting-summary-flat.pdf

Life sciences study design

All studies must disclose on these points even when the disclosure is negative.

Sample size	Protein crystals were mounted into cryo-loops with a size of 0.1-0.2 micrometer. Sample size was similar to the previous studies such as Reference #39 in the manuscript (Molecular Cell, 2019, PMID: 30639240). After several preliminary experiments, the authors chose 20µl or 30µl of total volumes for the cleavage assays, and certain concentrations of plasmids or oligonucleotide substrates were added into the total volumes of the cleavage assays, which could ensure the cleavage results to be clearly observed.
Data exclusions	No data was excluded.
Replication	Reproducibility of findings was established by performing crystallization protocol multiple times to ensure repeatability of crystallization conditions. After several preliminary experiments, total volumes for the cleavage assays, sample concentrations, reaction times and temperatures were determined. Each cleavage assay was independently carried out at least three times to verify the repeatability of the results. All attempts at replication were successful.
Randomization	Randomization was not applicable in the study design. The experimental results were reproducible stably and were not dependent on any conditions such as the batch of plasmid, oligonucleotide, RNA, protein, or solutions.
Blinding	Structure determinations were carried out without blinding, sample identity was known to the investigators.

Reporting for specific materials, systems and methods

We require information from authors about some types of materials, experimental systems and methods used in many studies. Here, indicate whether each material, system or method listed is relevant to your study. If you are not sure if a list item applies to your research, read the appropriate section before selecting a response.

Materials & experimental systems

n/a	Involved in the study	
☒	☐	Antibodies
☒	☐	Eukaryotic cell lines
☒	☐	Palaeontology and archaeology
☒	☐	Animals and other organisms
☒	☐	Human research participants
☒	☐	Clinical data
☒	☐	Dual use research of concern

Methods

n/a	Involved in the study	
☒	☐	ChIP-seq
☒	☐	Flow cytometry
☒	☐	MRI-based neuroimaging
